# Modulation of TNF-α Secretion in Peripheral Blood Mononuclear Cells by Cocoa Flavanols and Procyanidins

**DOI:** 10.1080/1044667031000137601

**Published:** 2002-09

**Authors:** T. K. Mao, J. van de Water, C. L. Keen, H. H. Schmitz, M. E. Gershwin

**Affiliations:** 1 Division of Rheumatology/Allergy and Clinical Immunology University of California at Davis School of Medicine TB 192, One Shields Avenue Davis CA 95616 USA; 2 Department of Nutrition University of California at Davis Davis CA 95616 USA; 3 Analytical and Applied Sciences Mars, Incorporated Hackettstown NJ 07840 USA

## Abstract

Epidemiological reports have suggested that the consumption of foods rich in flavonoids is associated with a lower incidence of certain degenerative diseases, including cardiovascular disease. Flavanols and their related oligomers, the procyanidins CFP, isolated from cocoa can modulate the production and level of several signaling molecules associated with immune function and inflammation *in vitro*, including several cytokines and eicosanoids. To further elucidate the potential immuno-modulatory functions of flavanol-rich cocoa, the present investigation examined whether isolated CFP fractions (monomers through decamers) influence the secretion of tumor necrosis factor-α (TNF-α) from resting and phytohemagluttinin (PHA)-stimulated human peripheral blood mononuclear cells (PBMC). We used an *in vitro* culture system where PBMC from 14 healthy subjects were introduced to individual CFP fractions for 72 h prior to measuring the levels of TNF-α released. The intermediate-sized CFP fractions (tetramers through octamers) were the most active on resting cells, causing a 3–4 fold increase in TNF-α relative to media baseline. The monomers and dimers were the least stimulatory of the fractions tested, displaying a 42 and 31% increase, respectively, over media control, whereas the trimers, nonamers and decamers showed an intermediate stimulation of this cytokine. In the presence of PHA, the intermediate-sized CFP fractions again were the most active, enhancing TNF-α secretion in the range of 48–128% relative to the PHA control. The monomers and dimers were slightly inhibitory (–1.5 and –15%, respectively), while trimers, nonamers and decamers stimulated moderate increases in TNF-α levels (13, 19 and 15%, respectively). The above results lend support to the concept that CFP can be immunomodulatory. The stimulation of TNF-α secretion may contribute to the putative beneficial effects of dietary flavanoids against microbial infection and tumorigenesis.


*Developmental Immunology*, September 2002, Vol. 9 (3), pp. 135–141

Taylor & Francis

Taylor & Francis Group

### Modulation of TNF-α Secretion in Peripheral Blood Mononuclear Cells by Cocoa Flavanols and Procyanidins

T.K. MAO[a], J. VAN DE WATER[a], C.L. KEEN[b], H.H. SCHMITZ[c] and M.E. GERSHWIN[a,*]

[a]*Division of Rheumatology/Allergy and Clinical Immunology, University of California at Davis, School of Medicine, TB 192, One Shields Avenue, Davis, CA 95616, USA;* [b]*Department of Nutrition, University of California at Davis, Davis, CA 95616, USA;* [c]*Analytical and Applied Sciences, Mars, Incorporated, Hackettstown, NJ 07840, USA*

Epidemiological reports have suggested that the consumption of foods rich in flavonoids is associated with a lower incidence of certain degenerative diseases, including cardiovascular disease. Flavanols and their related oligomers, the procyanidins CFP, isolated from cocoa can modulate the production and level of several signaling molecules associated with immune function and inflammation *in vitro*, including several cytokines and eicosanoids. To further elucidate the potential immuno-modulatory functions of flavanol-rich cocoa, the present investigation examined whether isolated CFP fractions (monomers through decamers) influence the secretion of tumor necrosis factor-α (TNF-α) from resting and phytohemagluttinin (PHA)-stimulated human peripheral blood mononuclear cells (PBMC). We used an *in vitro* culture system where PBMC from 14 healthy subjects were introduced to individual CFP fractions for 72 h prior to measuring the levels of TNF-α released. The intermediate-sized CFP fractions (tetramers through octamers) were the most active on resting cells, causing a 3–4 fold increase in TNF-α relative to media baseline. The monomers and dimers were the least stimulatory of the fractions tested, displaying a 42 and 31% increase, respectively, over media control, whereas the trimers, nonamers and decamers showed an intermediate stimulation of this cytokine. In the presence of PHA, the intermediate-sized CFP fractions again were the most active, enhancing TNF-α secretion in the range of 48–128% relative to the PHA control. The monomers and dimers were slightly inhibitory (−1.5 and −15%, respectively), while trimers, nonamers and decamers stimulated moderate increases in TNF-α levels (13, 19 and 15%, respectively). The above results lend support to the concept that CFP can be immunomodulatory. The stimulation of TNF-α secretion may contribute to the putative beneficial effects of dietary flavanoids against microbial infection and tumorigenesis.

*Keywords*: Monomers; Peripheral blood mononuclear cells; Tumor necrosis factor-α; Cocoa

### INTRODUCTION

Cocoa and its derivative products, such as chocolate, have recently garnered considerable scientific attention, in part because they can be rich in certain flavanoids. In particular, flavanol subclass, including (−)-epicatechin, (+)-catechin and their related oligomers, the procyanidins CFP, can be abundant in cocoa and chocolate products (Fig. 1) (Hammerstone *et al.*, 1999; Lazarus *et al.*, 1999). The presence of flavonoids in notable concentrations in certain foods (e.g. select fruits, vegetables and legumes) has triggered numerous studies on their potential health benefits, and accumulating epidemiological evidence suggests that consumption of flavonoid-rich foods and beverages are associated with a reduced risk of cardiovascular disease and certain cancers (Rice-Evans *et al.*, 1995; Stoner and Mukhtar, 1995; Holt *et al.* 2002a; Kris-Etherton and Keen, 2002). While cocoa contains a complex mixture of CFP, only the monomeric flavanols and small amounts of the procyanidin dimers have been measured in blood following consumption (Rein *et al.*, 2000; Wang *et al.*, 2000; Baba *et al.*, 2001; 2002; Holt *et al.*, 2002b; Steinberg *et al.*, 2002). Limited information is available concerning the absorption and metabolism of the larger cocoa procyanidins; however, a significant fraction of these procyanidins (≥ trimers) appear to reach the upper intestine intact following the consumption of a CFP-enriched meal (Rios *et al.*, 2002).

Recently, select CFP have demonstrated activity *in vivo* and *in vitro* that is suggestive of potential cardiovascular benefits. The focus of this research has generally centered around the concept that CFP-rich foods might improve tissue oxidant defense systems (Rice-Evans *et al.*, 1995; Kris-Etherton and Keen, 2002), reduce platelet reactivity

*Corresponding author. Tel.: +1-530-752-2884. Fax: +1-530-752-4669. E-mail: megershwin@ucdavis.edu

ISSN 1044-6672 print © 2002 Taylor & Francis Ltd
DOI: 10.1080/1044667031000137601

(Rein *et al.*, 2000; Holt *et al.*, 2002a; Pearson *et al.*, 2002) and improve vascular health by inducing vascular relaxation (Arteel and Sies, 1999; Stein *et al.*, 1999; Karim *et al.*, 2000; Duffy *et al.*, 2001). The mechanisms responsible, at least in part, for CFP-induced increases in endothelium-dependent relaxation are thought to involve the modulation of nitric oxide (NO) (Arteel and Sies, 1999; Karim *et al.*, 2000) and select eicosanoid levels (Schramm *et al.*, 2001; Holt *et al.*, 2002a; Schewe *et al.*, 2002), as well as the augmentation of select arms of the oxidant defense system (Rice-Evans *et al.*, 1995; 1996; Bagchi *et al.*, 1997; Virgili *et al.*, 1998; Arteel and Sies, 1999; Lotito *et al.*, 2000; Wang *et al.*, 2000; Pearson *et al.*, 2001; Kris-Etherton and Keen, 2002; Zhu *et al.*, 2002).

Complementing the above, we have demonstrated that distinct CFP fractions have the potential to modulate the production of several immunomodulatory cytokines. Under similar *in vitro* culture settings, select CFPs were shown to effectively suppress the production of cytokines (IL-1β and IL-2) that promote inflammation (Mao *et al.*, 1999; 2000a,b) while increasing the release of anti-inflammatory cytokines (IL-4 and IL-5) (Mao *et al.*, 2000c; 2002). Interestingly, CFP had a differential effect on the peripheral blood mononuclear cells (PBMC) production of TGF-$\beta_1$, depending on the constitutive production of the cytokine (Mao *et al.*, 2003). Tumor growth factor-$\beta_1$ can benefit the cardiovascular system by actively maintaining the normal physiological phenotype of endothelial cells and smooth muscle cells in the arterial vessel wall (Grainger, 1997). However, excess production of TGF-$\beta_1$ can cause extracellular matrix accumulation that is unfavorable for injured vessel wall, which can consequently lead to cardiac fibrosis (Lijnen *et al.*, 2000). We observed that the PBMC isolated from individuals with low baseline levels of TGF-$\beta_1$ were stimulated by individual fractions to enhance secretion, whereas production from PBMC isolated from high baseline producers was inhibited by the cocoa procyandins and flavanols (Mao *et al.*, 2003). The above results suggest that CFP can have differential effects in the production of certain cytokines depending on the immunological status of the individual. In a separate study, we reported that smaller CFPs may contribute to the health of the oral cavity by augmenting the mitogen-induced secretion of IL-5 (Mao *et al.*, 2002). Interleukin-5, as an integral cytokine, is responsible for mediating the production of IgA, and thus conferring protection against periodontitis (Kinane *et al.*, 1999; Salvi and Holgate, 1999). In the current investigation, we wanted to extend our existing knowledge of the immuno-modulatory potential of CFP to their effects on the production of tumor necrosis factor-alpha (TNF-α) from human PBMC.

### MATERIALS AND METHODS

### Cocoa Fraction Preparation

Water soluble CFP fractions were prepared from Cocoapro™ cocoa and quantified by LC-MS as detailed by Adamson *et al.* (1999), and were provided by Mars, Incorporated (Hackettstown, NJ). The monomers fraction contained the flavan-3-ols, (−)-epicatechin and (+) catechin. Purified oligomers (dimers through decamers) of these monomeric procyanidins were investigated (Fig. 1). The purified CFP fractions contained less than 0.5% (w/w) of total alkaloids (theobromine and caffeine). The monomer and procyanidin composition, estimated by HPLC and molecular weights of these preparations are shown in Table I. All samples were suspended in RPMI 1640 (Invitrogen Corp., Carlsbad, CA) with 10% heat inactivated fetal bovine serum (Atlanta Biologicals, Norcross, GA). They were then diluted with the same medium to final concentrations of

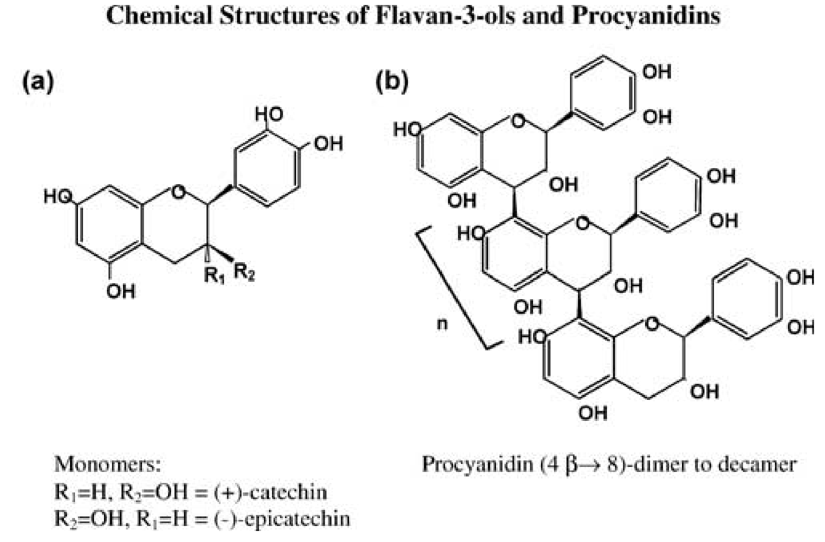

FIGURE 1 Chemical structures of the most common flavan-3-ol monomers and procyanidins (dimers to decamers) in cocoa.

TABLE I Profile of the individual cocoa FP fractions

| Fraction name | Molecular weight (Da) | Procyanidin profile | % |
|---|---|---|---|
| Monomers | 290 | Monomers | 95 |
| Dimers | 578 | Dimers | 98 |
| Trimers | 866 | Trimers | 93 |
| Tetramers | 1154 | Tetramers | 93 |
| Pentamers | 1442 | Pentamers | 93 |
| Hexamers | 1730 | Hexamers | 89 |
| Heptamers | 2018 | Heptamers | 79 |
| | | Hexamers | 18 |
| Octamers | 2306 | Octamers | 76 |
| | | Heptamers | 16 |
| Nonamers | 2594 | Nonamers | 60 |
| | | Octamers | 28 |
| Decamers | 2882 | Decamers | 40 |
| | | Nonamers | 17 |
| | | Octamers | 22 |
| | | Heptamers | 16 |

25 μg/ml. We based the concentration of flavonoids used in this study on prior dose response analysis conducted by our laboratory and others assessing the impact of these compounds on cytokine production and release (Sanbongi *et al.*, 1997; Mao *et al.*, 1999; 2000a,b,c; 2002).

### PBMC Isolation

Peripheral blood from healthy volunteers was collected into sodium citrate-containing tubes and mixed 1:1 with Hanks' Balanced Salt Solution (HBSS; Invitrogen) without calcium chloride, magnesium chloride or magnesium sulfate. The diluted blood was then layered over a Histopaque®-1077 gradient (Sigma, St Louis, MO) and centrifuged at 500*g* for 30 min at room temperature. PBMC were harvested from the interface layer, washed twice with HBSS and then counted. The cells were resuspended in RPMI 1640 containing 10% fetal bovine serum and supplemented with 0.1% of a 50 mg/ml gentamicin solution (Invitrogen). PBMC concentration was adjusted to $2 \times 10^6$ viable cells/ml after estimation of viability by trypan blue exclusion assay. Viability was consistently greater that 96%.

### Culture of PBMC with Cocoa FP Fractions

Five hundred microliters of a $1.0 \times 10^6$ cell suspension were cultured with equal volumes of the various CFP treatments at 37°C with 5% $CO_2$ in 48-well plates. Resting PBMC were incubated with individual CFP fractions at 25 μg/ml in the presence or absence of PHA at 25 μg/ml. All treatments were performed in duplicate. Following a 72 h incubation, the supernatant fractions were harvested for ELISA analysis.

### TNF-α (ELISA)

Culture supernatant fractions were harvested after 72 h and stored at −20°C until analysis by ELISA. Ninety-six well Costar EIA plates (Cat. #2592) were coated with mouse anti-TNF-α supplied in the DuoSet Human TNF-α ELISA Development Kit (R and D Systems, Minneapolis, MN). Supernates were measured for TNF-α concentrations according to the manufacturers' recommendations. The lowest TNF-α standard for the ELISA system was 15.6 pg/ml.

### Statistics

The effects of different CFP fractions on the secretion of TNF-α were examined in unstimulated resting PBMC. Results were compared by Student paired *t*-test with a two-tailed *p*-value (i.e. control cells without CFP vs. cells treated with individual CFP fractions). Significance was taken as $p < 0.05$.

### RESULTS

Unstimulated resting PBMC were prepared and incubated with individual CFP fractions at 25 μg/ml. TNF-α production was then assessed in the supernatant fractions following a 72 h incubation period. ELISA analysis revealed that the CFP were stimulatory for the secretion of TNF-α, regardless of the degree of oligomerization (Fig. 2). The monomeric and dimeric fractions were the least stimulatory of the group of fractions tested, displaying 42 and 31% increases, respectively, over media baseline level (315 ± 96 pg/ml) (Table II). The intermediate-sized procyanidins (tetramers through octamers) induced a marked 3–4 fold increase over media baseline (Table II). At levels between the two extremes, the trimeric, nonumeric and decameric fractions stimulated the release of TNF-α approximately two fold over baseline control (Table II).

In the second part of this study, the effects of CFP on the production of TNF-α by stimulated PBMC were investigated. In a similar *in vitro* culture system, the CFP treated cells, at the same concentration (25 μg/ml) as described above, were co-incubated with PHA at 25 μg/ml for 72 h prior to the harvest of supernates. Phytohemagglutinin, a mitogen known to stimulate $CD4^+$ $T_H1$ and $CD8^+$ T cells, caused a marked increase in TNF-α secretion (2668 ± 318 pg/ml) from PBMC in comparison to unstimulated media control (315 ± 96 pg/ml). In PHA-stimulated PBMC, the CFP fractions displayed similar patterns of response to that observed in the resting cells (Fig. 3). In particular, the intermediate-sized fractions (tetramers through octamers) were able to significantly enhance the level of TNF-α secretion in the range of 48–128% over the PHA control (Table II). Consistent with the CFP effects observed in resting PBMC, the trimeric, nonumeric and decameric fractions induced moderate increases of 13–19% over the PHA control in stimulated cells. In contrast to the higher molecular weight fractions, the monomeric and dimeric cocoa fractions suppressed the PHA-induced response by 1.5 and 15%, respectively.

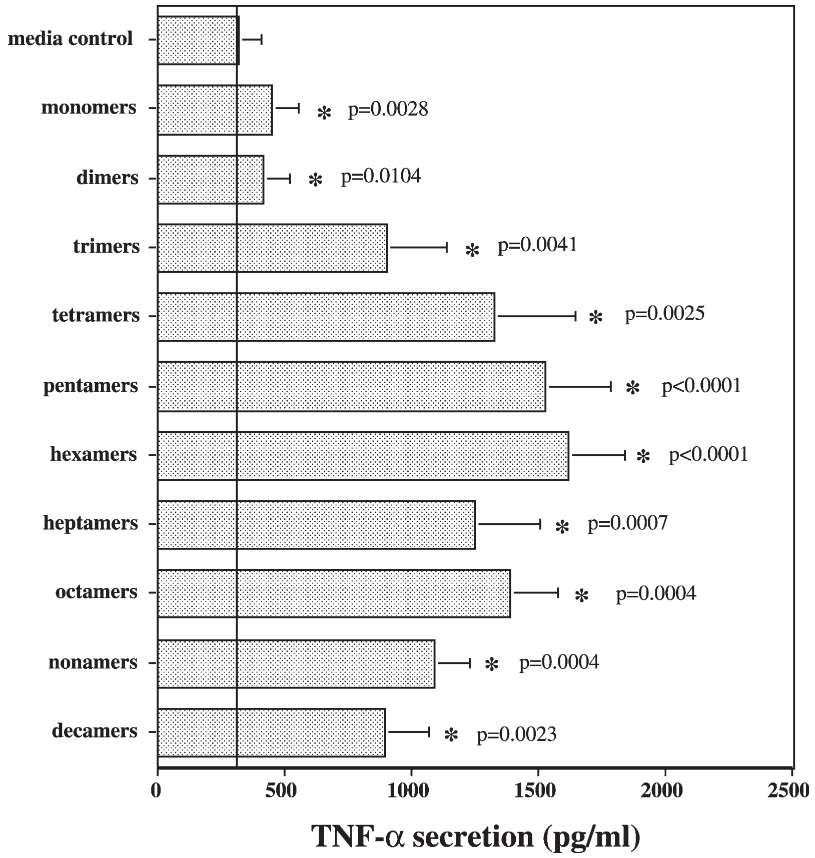

FIGURE 2 The effect of cocoa FP on secretion of TNF-α from unstimulated PBMC. PBMC were incubated with individual cocoa fractions (25 μg/ml) for 72 h before supernates were extracted for ELISA analysis (mean ± SEM; $n = 10$). Values induced from cocoa treatment were compared with control values (i.e. media baseline without cocoa) using a Student paired *t*-test with a two-tailed *p*-value. *Significance was taken as $p < 0.05$.

### DISCUSSION

The appealing association of a diet rich in flavanols with a variety of health benefits has sparked numerous studies to corroborate the epidemiological findings. A number of investigations have shown that flavanols, including those derived from cocoa seeds, display antioxidant activities (Rice-Evans *et al.*, 1995; Rice-Evans *et al.*, 1996; Bagchi *et al.*, 1997; Virgili *et al.*, 1998; Pearson *et al.*, 2001; Kris-Etherton and Keen, 2002), as well as the ability to modulate the levels of eicosanoids, NO and peroxynitrite (Arteel and Sies, 1999; Schramm *et al.*, 2001; Schewe *et al.*, 2002). Complementary to the properties suggested above, we have demonstrated that select CFP fractions can modulate the production of cytokines associated with inflammation (IL-1β, IL-2 and IL-4) (Mao *et al.*, 1999; 2000a,b,c), vascular health (TGF-$\beta_1$) (Mao *et al.*, 2003), and oral cavity health (IL-5) (Mao *et al.*, 2002). Here, we have extended these findings to evaluate the effect of CFP monomers to decamers on the production of TNF-α.

TABLE II Effect of cocoa FP fractions on TNF-α secretion in unstimulated ($n = 10$) and PHA-stimulated ($n = 14$) PBMCs. In each group, the mean values from cocoa treated samples are compared to corresponding mean baseline values and expressed as percent change from media and PHA baseline controls. The levels of TNF-α secretion in pg/ml are indicated in parenthesis (mean ± SEM)

| FP fraction | Unstimulated PBMCs | PHA-stimulated PBMCs |
|---|---|---|
| Controls | Media (315 ± 96) | PHA (2668 ± 318) |
| Monomers | + 42% (447 ± 109) | − 1.5% (2628 ± 289) |
| Dimers | + 31% (414 ± 112) | − 15% (2259 ± 264) |
| Trimers | + 184% (895 ± 243) | + 13% (3028 ± 471) |
| Tetramers | + 320% (1324 ± 326) | + 64% (4382 ± 612) |
| Pentamers | + 384% (1525 ± 262) | + 116% (5774 ± 725) |
| Hexamers | + 412% (1614 ± 227) | + 128% (6086 ± 783) |
| Heptamers | + 295% (1243 ± 267) | + 61% (4292 ± 676) |
| Octamers | + 338% (1381 ± 199) | + 48% (3952 ± 522) |
| Nonamers | + 244% (1083 ± 146) | + 19% (3166 ± 384) |
| Decamers | + 183% (890 ± 179) | + 15% (3080 ± 490) |

In the present study, we have shown that the intermediate-sized CFP fractions (tetramers to octamers) can significantly augment the release of TNF-α from unstimulated, and mitogen-induced human PBMC, while the monomeric and dimeric fractions proved to be only moderately stimulatory in resting cells, and slightly inhibitory in the presence of PHA. It has been previously shown that mitogen-stimulated human PBMC displayed optimum TNF-α levels at 24 h, and that the levels steadily decline following this timepoint (Feldman *et al.*, 1999). Since our measurements were taken at 72 h post-exposure, it is possible that the levels observed with our current protocol are significantly diminished. Indeed, the inhibition displayed by the monomeric and dimeric CFP fractions with PHA-stimulated PBMC might be

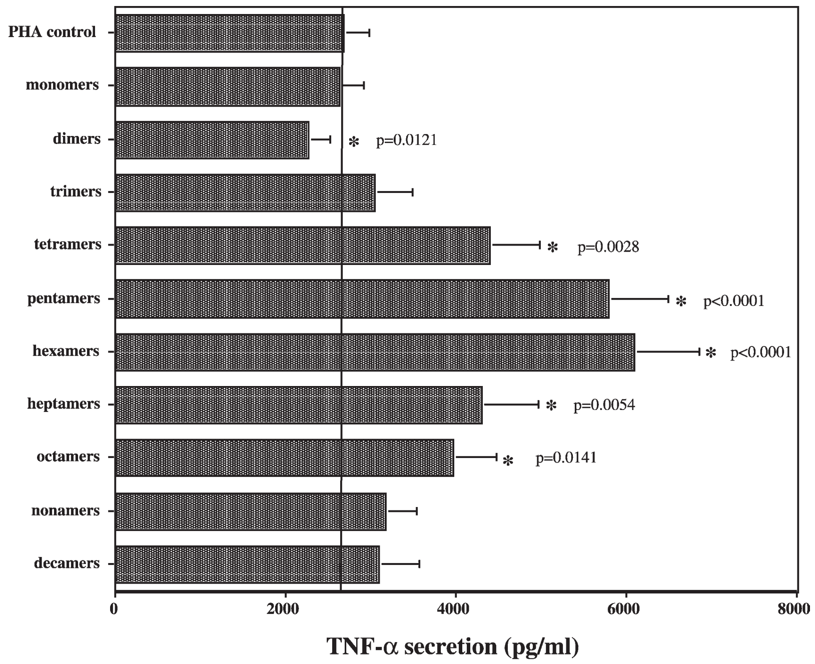

FIGURE 3 The effect of cocoa FP on secretion of TNF-α from PHA-stimulated PBMC. PBMC were incubated with individual cocoa fractions (25 μg/ml) in the presence of PHA (25 μg/ml) for 72 h before supernates were extracted for ELISA analysis (mean ± SEM; $n = 14$). Values induced from cocoa treatment were compared with control values (i.e. media baseline without cocoa) using a Student paired *t*-test with a two-tailed *p*-value. *Significance was taken as $p < 0.05$.

misleading, as these small fractions might also augment TNF-α secretion during early exposure.

Although flavanols, a subgroup of compounds belonging to the flavanoid family, are found in both cocoa and tea, there are significant structural differences, not to mention the catechin content of chocolate can be four times that of tea (Arts *et al.*, 1999). The predominant catechins found in tea are (−)-epicatechin gallate and (−)-epigallocatechin gallate (EGCG), with EGCG accounting for greater than 40% of the total polyphenols (Hara, 1997). Several groups have reported that EGCG can inhibit TNF-α production at both the transcriptional and protein levels by blocking nuclear transcription factor-κB (NF-κB) in rat and murine models (Yang *et al.*, 1998; Fujiki *et al.*, 1999; Suganuma *et al.*, 2000; Yang *et al.*, 2001). Interestingly, when EGCG was added to a murine alveolar macrophage cell line (MH-S) pretreated with nicotine, EGCG diminished the nicotine-induced suppression of TNF-α production (Matsunaga *et al.*, 2002). However, in another study utilizing human leukocytes, EGCG had no effect on the production of TNF-α (Crouvezier *et al.*, 2001).

TNF-α is a proinflammatory cytokine with numerous biological functions critical for response to infections. A pivotal role of TNF-α is its ability to activate, and subsequently to enhance, the antimicrobial and tumoricidal activities of macrophages. In a previous study, we reported that the pentameric through decameric CFP fractions can be stimulatory for another proinflammatory cytokine, IL-1β, in the presence and absence of PHA (Mao *et al.*, 2000a). Taken together with our present study, it is evident that under some circumstances, the larger cocoa CFP fractions (≥ pentamer) can be proinflammatory, triggering high production of IL-1β and TNF-α relative to controls.

The mechanism(s) by which CFP modulate cytokine production are unclear. However, eicosanoids have been postulated to play a pivotal role in the regulation of TNF-α gene expression (Jongeneel, 1994). Prostaglandins and leukotrienes are known to have antagonistic effects with the downregulation of TNF-α production attributed to prostaglandins (Jongeneel, 1994). Inhibition of cyclooxygenase (COX) and lipoxygenase, key enzymes in the biosythesis of prostaglandins and leukotrienes, respectively, will in turn alter TNF-α levels (Jongeneel, 1994). Recently, it has been shown that (+)-catechin can inhibit COX activity (Noreen *et al.*, 1998). Therefore, it is plausible that the TNF-α stimulation observed with our monomeric fraction is, in part, due to the presence of (+)-catechin interfering with the normal negative feedback loop mediated by prostaglandins (Jongeneel, 1994). Alternatively, it has recently been shown that CFP, particularly (−)-epicatechin and the procyanidin dimers, can display significant inhibition of 5-lipoxygenase, whereas the trimeric through pentameric fractions show intermediate suppression, and the larger procyanidins (hexamers through nonamers) are relatively inactive (Schewe *et al.*, 2002). This observation is consistent with the present analysis in which the monomer and dimer fractions were the least stimulatory when compared with the larger procyanidins.

As alluded to above, there is currently no consensus concerning the effects of flavonoids on the production of

TNF-α. In one study, it was shown that monomeric and dimeric procyanidins inhibited TNF-α release from murine RAW 264.7 macrophages, while the trimeric fraction enhanced secretion (Park *et al.*, 2000). Wine polyphenols also displayed different effects on cytokine production. Whereas quercetin decreased the LPS-stimulated release of TNF-α from RAW 264.7 macrophages, resveratrol augmented production (Wadsworth and Koop, 1999). Clearly, the discrepancy in these *in vitro* studies lies in the structure of plant polyphenols and their degree of polymerization, as well as the type of cells that are being exposed to these compounds in an *in vitro* setting. Nevertheless, our analysis suggests that incubation of human PBMC with CFP fractions not only modulates the production of TNF-α, but can significantly upregulate the synthesis and secretion of this pro-inflammatory cytokine. In summary, this investigation implicates that CFP may provide thearapeutic protection against certain microbial infections and tumorigenesis. Given the bioavailability of the CFP compounds, it can be speculated that the higher oligomers will primarily exert effects in the gastrointestinal tract. The relatively moderate effect of the monomers and dimers on TNF-α production suggests that they would have minimal effects on cardiovascular health with respect to TNF-α pathways.

### Acknowledgements

This work was supported by a grant from Mars, Incorporated.

### References

Adamson, G.E., Lazarus, S.A., Mitchell, A.E., Prior, R.L., Cao, G., Jacobs, P.H., Kremers, B.G., Hammerstone, J.F., Rucker, R.B., Ritter, K.A. and Schmitz, H.H. (1999) "HPLC method for the quantification of procyanidins in cocoa and chocolate samples and correlation to total antioxidant capacity", *J. Agric. Food Chem.* **47**, 4184–4188.

Arteel, G.E. and Sies, H. (1999) "Protection against peroxynitrite by cocoa polyphenol oligomers", *FEBS Lett.* **462**, 167–170.

Arts, I.C.W., Hollman, P.C.H. and Kromhout, D. (1999) "Chocolate as a source of tea flavonoids", *Lancet* **354**, 488.

Baba, S., Osakabe, N., Natsume, M., Muto, Y., Takizawa, T. and Terao, J. (2001) "Absorption and urinary excretion of (−)-epicatechin after administration of different levels of cocoa powder or (−)-epicatechin in rats", *J. Agric. Food Chem.* **49**, 6050–6056.

Baba, S., Osakabe, N., Natsume, M. and Terao, J. (2002) "Absorption and urinary excretion of procyanidin B2 [epicatechin-(4beta-8)-epicatechin] in rats", *Free Radic. Biol. Med.* **33**, 142–148.

Bagchi, D., Garg, A., Krohn, R.L., Bagchi, M., Tran, M.X. and Stohs, S.J. (1997) "Oxygen free radical scavenging abilities of vitamins C and E, and a grape seed proanthocyanidin extract *in vitro*", *Res. Commun. Mol. Pathol. Pharmacol.* **95**, 179–189.

Crouvezier, S., Powell, B., Keir, D. and Yaqoob, P. (2001) "The effects of phenolic components of tea on the production of pro- and anti-inflammatory cytokines by human leukocytes *in vitro*", *Cytokine* **13**, 280–286.

Duffy, S.J., Keaney, J.F. Jr, Holbrook, M., Gokce, N., Swerdloff, P.L., Frei, B. and Vita, J.A. (2001) "Short- and long-term black tea consumption reverses endothelial dysfunction in patients with coronary artery disease", *Circulation* **104**, 151–156.

Feldman, K.S., Sahasrabudhe, K., Smith, R.S. and Scheuchenzuber, W.J. (1999) "Immunostimulation by plant polyphenols: a relationship between tumor necrosis factor-alpha production and tannin structure", *Bioorg. Med. Chem. Lett.* **9**, 985–990.

Fujiki, H., Suganuma, M., Okabe, S., Sueoka, E., Suga, K., Imai, K., Nakachi, K. and Kimura, S. (1999) "Mechanistic findings of green tea as cancer preventive for humans", *Proc. Soc. Exp. Biol. Med.* **220**, 225–228.

Grainger, D.J. (1997) "Transforming growth factor-beta and cardiovascular protection", The Endothelium in Clinical Practice (Marcel Dekker, Inc., New York), pp 203–243.

Hammerstone, J.F. Jr, Lazarus, S.A., Mitchell, A.E., Rucker, R. and Schmitz, H.H. (1999) "Identification of procyanidins in cocoa (*Theobroma cacao*) and chocolate using high-performance liquid chromatography/mass spectrometry", *J. Agric. Food Chem.* **47**, 490–496.

Hara, Y. (1997) "Influence of tea catechins on the digestive tract", *J. Cell. Biochem.* **Suppl. 27**, 52–58.

Holt, R.R., Schramm, D.P., Keen, C.L., Lazarus, S.A. and Schmitz, H.H. (2002a) "Chocolate consumption and platelet function", *J. Am. Med. Assoc.* **287**, 2212–2213.

Holt, R.R., Lazarus, S.A., Sullards, M.C., Zhu, Q.Y., Schramm, D.D., Hammerstone, J.F., Fraga, C.G., Schmitz, H.H. and Keen, C.L. (2002b) "Procyanidin dimer B2 [epicatechin-(4beta-8)-epicatechin] in human plasma after the consumption of a flavanol-rich cocoa", *Am. J. Clin. Nutr.* **76**, 798–804.

Jongeneel, C.V. (1994) "Regulation of the TNF alpha gene", *Prog. Clin. Biol. Res.* **388**, 367–381.

Karim, M., McCormick, K. and Kappagoda, C.T. (2000) "Effects of cocoa extracts on endothelium-dependent relaxation", *J. Nutr.* **130**, 2105S–2108S.

Kinane, D.F., Lappin, D.F., Koulouri, O. and Buckley, A. (1999) "Humoral immune responses in periodontal disease may have mucosal and systemic immune features", *Clin. Exp. Immunol.* **115**, 534–541.

Kris-Etherton, P.M. and Keen, C.L. (2002) "Evidence that the antioxidant flavonoids in tea and cocoa are beneficial for cardiovascular health", *Curr. Opin. Lipidol.* **13**, 41–49.

Lazarus, S.A., Hammerstone, J.F. and Schmitz, H.H. (1999) "Chocolate contains additional flavonoids not found in tea", *Lancet* **354**, 1825.

Lijnen, P.J., Petrov, V.V. and Fagard, R.H. (2000) "Induction of cardiac fibrosis by transforming growth factor-beta(1)", *Mol. Genet. Metab.* **71**, 418–435.

Lotito, S.B., Actis-Goretta, L., Renart, M.L., Caligiuri, M., Rein, D., Schmitz, H.H., Steinberg, F.M., Keen, C.L. and Fraga, C.G. (2000) "Influence of oligomer chain length on the antioxidant activity of procyanidins", *Biochem. Biophys. Res. Commun.* **276**, 945–951.

Mao, T.K., Powell, J.J., van de Water, J., Keen, C.L., Schmitz, H.H. and Gershwin, M.E. (1999) "The influence of cocoa procyanidins on the transcription of interleukin-2 in peripheral blood mononuclear cells", *Int. J. Immunother.* **15**, 23–29.

Mao, T.K., Powell, J.J., van de Water, J., Keen, C.L., Schmitz, H.H., Hammerstone, J.F. and Gershwin, M.E. (2000a) "The effect of cocoa procyanidins on the transcription and secretion of interleukin 1b in peripheral blood mononuclear cells", *Life Sci.* **66**, 1377–1386.

Mao, T., van de Water, J., Keen, C.L., Schmitz, H.H. and Gershwin, M.E. (2000b) "Cocoa procyanidins and human cytokine transcription and secretion", *J. Nutr.* **130**, 2093S–2099S.

Mao, T.K., Powell, J.J., van de Water, J., Keen, C.L., Schmitz, H.H. and Gershwin, M.E. (2000c) "Effect of cocoa procyanidins on secretion of interleukin-4 in peripheral blood mononuclear cells", *J. Med. Foods* **3**, 107–114.

Mao, T.K., van de Water, J., Keen, C.L., Schmitz, H.H. and Gershwin, M.E. (2002) "Effect of cocoa flavanols and their related oligomers on the secretion of interleukin-5 in peripheral blood mononuclear cells", *J. Med. Foods* **5**, 17–22.

Mao, T.K., van de Water, J., Keen, C.L., Schmitz, H.H. and Gershwin, M.E. (2003) "Cocoa flavonols and procyanidins promote transforming growth factor-beta1 homeostasis in peripheral blood mononuclear cells", *Exp. Biol. Med. (Maywood)* **228**, 93–99.

Matsunaga, K., Klein, T.W., Friedman, H. and Yamamoto, Y. (2002) "*In vitro* therapeutic effect of epigallocatechin gallate on nicotine-induced impairment of resistance to Legionella pneumophila infection of established MH-S alveolar macrophages", *J. Infect. Dis.* **185**, 229–236.

Noreen, Y., Ringbom, T., Perera, P., Danielson, H. and Bohlin, L. (1998) "Development of a radiochemical cyclooxygenase-1 and -2 *in vitro* assay for identification of natural products as inhibitors of prostaglandin biosynthesis", *J. Nat. Prod.* **61**, 2–7.

Park, Y.C., Rimbach, G., Saliou, C., Valacchi, G. and Packer, L. (2000) "Activity of monomeric, dimeric, and trimeric flavonoids on NO production, TNF-alpha secretion and NF-kappaB-dependent gene expression in RAW 264.7 macrophages", *FEBS Lett.* **465**, 93–97.

Pearson, D.A., Schmitz, H.H., Lazarus, S.A. and Keen, C.L. (2001) "Inhibition of *in vitro* low-density lipoprotein oxidation by oligomeric procyanidins present in chocolate and cocoas", *Methods Enzymol.* **335**, 350–360.

Pearson, D.A., Paglieroni, T.G., Rein, D., Wun, T., Schramm, D.D., Wang, J.F., Holt, R.R., Gosselin, R., Schmitz, H.H. and Keen, C.L. (2002) "The effects of flavonol-rich cocoa and aspirin on *ex vivo* platelet function", *Thromb. Res.* (in press).

Rein, D., Paglieroni, T.G., Wun, T., Pearson, D.A., Schmitz, H.H., Gosselin, R. and Keen, C.L. (2000) "Cocoa inhibits platelet activation and function", *Am. J. Clin. Nutr.* **72**, 30–35.

Rice-Evans, C.A., Miller, N.J., Bolwell, P.G., Bramley, P.M. and Pridham, J.B. (1995) "The relative antioxidant activities of plant-derived polyphenolic flavonoids", *Free Radic. Res.* **22**, 375–383.

Rice-Evans, C.A., Miller, N.J. and Paganga, G. (1996) "Structure-antioxidant activity relationships of flavonoids and phenolic acids", *Free Radic. Biol. Med.* **20**, 933–956.

Rios, L.Y., Bennett, R.N., Lazarus, S.A., Remesy, C., Scalbert, A. and Williamson, G. (2002) "Cocoa procyanidins are stable during gastric transit in humans", *Am. J. Clin. Nutr.* **76**, 1106–1110.

Salvi, S. and Holgate, S.T. (1999) "Could the airway epithelium play an important role in mucosal immunoglobulin A production?", *Clin. Exp. Allergy* **29**, 1597–1605.

Sanbongi, C., Suzuki, N. and Sakane, T. (1997) "Polyphenols in chocolate, which have antioxidant activity, modulate immune function in humans *in vitro*", *Cell. Immunol.* **177**, 129–136.

Schewe, T., Kuhn, H. and Sies, H. (2002) "Flavonoids of cocoa inhibit recombinant human 5-lipoxygenase", *J. Nutr.* **132**, 1825–1829.

Schramm, D.D., Wang, J.F., Holt, R.R., Ensunsa, J.L., Gonsalves, J.L., Lazarus, S.A., Schmitz, H.H., German, J.B. and Keen, C.L. (2001) "Chocolate procyanidins decrease the leukotriene-prostacyclin ratio in humans and human aortic endothelial cells", *Am. J. Clin. Nutr.* **73**, 36–40.

Stein, J.H., Keevil, J.G., Wiebe, D.A., Aeschlimann, S. and Folts, J.D. (1999) "Purple grape juice improves endothelial function and reduces the susceptibility of LDL cholesterol to oxidation in patients with coronary artery disease", *Circulation* **100**, 1050–1055.

Steinberg, F.M., Holt, R.R., Schmitz, H.H. and Keen, C.L. (2002) "Cocoa procyanidin chain length does not determine ability to protect LDL from oxidation when monomer units are controlled", *J. Nutr. Biochem.* **13**, 645–652.

Stoner, G.D. and Mukhtar, H. (1995) "Polyphenols as cancer chemopreventive agents", *J. Cell. Biochem. Suppl.* **22**, 169–180.

Suganuma, M., Sueoka, E., Sueoka, N., Okabe, S. and Fujiki, H. (2000) "Mechanisms of cancer prevention by tea polyphenols based on inhibition of TNF-alpha expression", *Biofactors* **13**, 67–72.

Virgili, F., Kobuchi, H. and Packer, L. (1998) "Procyanidins extracted from Pinus maritima (Pycnogenol): scavengers of free radical species and modulators of nitrogen monoxide metabolism in activated murine RAW 264.7 macrophages", *Free Radic. Biol. Med.* **24**, 1120–1129.

Wadsworth, T.L. and Koop, D.R. (1999) "Effects of the wine polyphenolics quercetin and resveratrol on pro-inflammatory cytokine expression in RAW 264.7 macrophages", *Biochem. Pharmacol.* **57**, 941–949.

Wang, J.F., Schramm, D.D., Holt, R.R., Ensunsa, J.L., Fraga, C.G., Schmitz, H.H. and Keen, C.L. (2000) "A dose-response effect from chocolate consumption on plasma epicatechin and oxidative damage", *J. Nutr.* **130**, 2115S–2119S.

Yang, F., de Villiers, W.J., McClain, C.J. and Varilek, G.W. (1998) "Green tea polyphenols block endotoxin-induced tumor necrosis factor-production and lethality in a murine model", *J. Nutr.* **128**, 2334–2340.

Yang, F., Oz, H.S., Barve, S., de Villiers, W.J., McClain, C.J. and Varilek, G.W. (2001) "The green tea polyphenol (−)-epigallocatechin-3-gallate blocks nuclear factor-kappa B activation by inhibiting I kappa B kinase activity in the intestinal epithelial cell line IEC-6", *Mol. Pharmacol.* **60**, 528–533.

Zhu, Q.Y., Holt, R.R., Lazarus, S.A., Orozco, T.J. and Keen, C.L. (2002) "Inhibitory effects of cocoa flavanols and procyanidin oligomers on free radical-induced erythrocyte hemolysis", *Exp. Biol. Med.* **227**, 321–329.

